# Cross-Sectional Study to Estimate the Prevalence of Inner-Ear Anomalies in Children With Congenital Sensorineural Hearing Loss by High-Resolution Computed Tomography (HRCT) Temporal Bone Scan

**DOI:** 10.7759/cureus.42160

**Published:** 2023-07-19

**Authors:** Deeksha D D, GC Patil

**Affiliations:** 1 Otolaryngology–Head and Neck Surgery, Karnataka Institute of Medical Sciences, Hubballi, IND; 2 Radiology, Karnataka Institute of Medical Sciences, Hubballi, IND

**Keywords:** children, prevalence, cochlea, inner ear, sensorineural, hearing loss

## Abstract

Background

Congenital sensorineural hearing loss (SNHL) is one of the most common birth defects with an incidence ratio of 1:1000 live births in India. Imaging plays an important role in the evaluation of congenital SNHL. As there is a paucity of studies in the Indian setting to determine the prevalence of inner-ear abnormalities, this study attempts to throw light on the various inner-ear anomalies that are prevalent in our setup in the Northern part of Karnataka using high-resolution computed tomography (HRCT) temporal bone scan.

Objectives

The objectives of this study are estimation of the prevalence of inner-ear anomalies in children with congenital SNHL by employing a radiologic assessment of HRCT temporal bone scans and determination of the factors associated with the identification of these abnormalities like demographic factors and degree of hearing loss.

Methods

Children with congenital SNHL underwent clinical evaluation with history taking and general and ear examination. Otoacoustic emission (OAE) and brainstem evoked response audiometry (BERA) measurements were obtained.

A radiological assessment by HRCT temporal bone scan was done. Using the classiﬁcation criteria of inner-ear malformations by Jackler and Sennaroglu as a reference, diagnostic standards were established in studying inner-ear malformations. Data were collected and entered in a Performa, which includes patient’s demography, audiological findings, and radiological findings, and the results were analyzed. Data were entered into Microsoft Excel, and statistical analysis was carried out using IBM SPSS Statistics for Windows, Version 27 (Released 2020; IBM Corp., Armonk, New York, United States). Categorical variables were presented as frequency and percentage. Then the prevalence of inner-ear anomaly was estimated. Correlation between inner-ear anomaly and other factors was calculated using the Chi-square test.

Results

The prevalence of inner-ear anomalies identified in congenital SNHL by HRCT scan was as follows: 26.08% (12/46), 26.1% (24/92) of inner ears was anomalous, 23.9% of the cochlea was anomalous, 6.5% of the vestibule was anomalous, 5.4% of the vestibular aqueduct was anomalous, and 3.2% of the semicircular canal was anomalous.

Cochlear aplasia, incomplete partition, common cavity, and cochlear hypoplasia were the anomalies found. Few cochleas had an abnormal cochlear height, though they appeared normal structurally. The dilated vestibule was the most common vestibular abnormality. There was a negative association found between the inner-ear anomaly in children with congenital SNHL who had a history of consanguineous marriage in their parents.

Conclusion

High-resolution temporal CT scanning could provide detailed information on the pathology of the inner ear in congenital SNHL, which can help in better planning the surgery for cochlear implantation and understanding the prognosis.

## Introduction

Congenital sensorineural hearing loss (SNHL) is one of the most common birth defects with an incidence ratio of 1:1000 live births in India [1]. Imaging plays an important role in the evaluation of congenital SNHL [2], and the cochlear implant has emerged as the rehabilitative option of choice for patients with severe to profound SNHL. However, candidacy determination for cochlear implants is a complex process, requiring careful consideration from an interdisciplinary team of professionals [3]. In children who are candidates for cochlear implantation surgery, imaging by high-resolution computed tomography (HRCT) temporal bone scans provides vital preoperative information about the inner ear [4]. It enables to identify anomalies that could complicate implant use. Approximately 20% of patients with congenital SNHL have radiographic abnormalities of the inner ear [5]. Vestibulocochlear dysplasia can assume a variety of forms, many of which are relevant to the surgical technique and postoperative performance. Moreover, cochlear aplasia is a noteworthy finding given that the absence of a cochlea is an absolute contraindication to cochlear implantation. Thus, when anomalies are recognized in the preoperative setting, patient counseling is vitally important. Moreover, the surgical team may need to consider device and technique alterations to account for anomalous anatomy. Inner-ear anomalies can also be associated with concomitant abnormalities like an aberrant coarse of the facial nerve [3]. Comprehensive evaluation of imaging studies is of important significance in determining the suitable ear for cochlear implants, evaluating electrode placement, and predicting intraoperative and postoperative complications. Therefore, determining the underlying pathology through preoperative examination is required to formulate the best approach for cochlear implantation surgery, and to achieve optimal results of rehabilitation [6].

In the northern part of Karnataka where this study is being conducted, there are children who need cochlear implants, although the prevalence of inner-ear anomalies is not known. There is a paucity of studies in the Indian setting to determine the prevalence of inner-ear anomalies, and for this reason, we decided to conduct this study in the Indian context to evaluate the various inner-ear anomalies by HRCT scan of the temporal bone.

The aim of this research is to study the distribution of different inner-ear anomalies in children with congenital SNHL. The primary objective is to estimate the prevalence of anomalous inner ear in children with congenital SNHL by radiologic assessment of HRCT temporal bone scans. The secondary objective is to determine the factors associated with identification of these anomalies, like demographic factors and the degree of hearing loss.

## Materials and methods

This study was conducted among children with congenital SNHL attending the outpatient department at Karnataka Institute of Medical Science, Hubballi, which is a government setup, over a period of two years from March 2021 to February 2023. Karnataka Institute of Medical Sciences, Hubballi Ethics Committee (Reg. No. ECR/486/Inst/KA/2013/RR-16) issued approval. Informed written valid consent was taken from the parents of all the recruited cases.

Inclusion criteria

Children with congenital SNHL diagnosed with OAE and BERA were included in the study.

Exclusion criteria

Patients with congenital conductive hearing loss, congenital cholesteatoma, and hearing loss due to an acquired cause like chronic otitis media are excluded.

Children with congenital SNHL underwent clinical evaluation with history taking and general and ear examination. Clinical history included age at identification of hearing loss, prenatal, natal, and postnatal history, mother’s drug intake and radiation exposure during pregnancy, mother’s history of fever during pregnancy, type of delivery and duration of hospitalization, birth weight, history of NICU admission, history of neonatal jaundice, developmental history, hearing aid use, family history of hearing loss, and neurological disorders.

The examination of the patient is inclusive of a general examination to look for dysmorphism and an ear, nose, and throat examination. The OAE and BERA measurements were obtained. The radiological assessment by HRCT temporal bone was done to see the status of morphology of the cochlea, cochlear height, cochlear duct length, vestibule, semicircular canal, and vestibular aqueduct. The scan parameters were 120 kV, 100 mA, thin-slice 0.6mm cuts HRCT.

As sedation was often necessary, the examination was done with the assistance of pediatricians. Triclofos 5 mL = 500 mg was given with a dosage of 50-70 mg/kg of body weight. An additional 5 ml was given after an hour if still not sedated.

CT scans are subjected to bone review programs for the enhancement of fine bony detail. Both axial and coronal views are studied. All images were evaluated in the RadiAnt DICOM viewer.

Using the classiﬁcation criteria of inner-ear malformations by Jackler and Sennaroglu as a reference, diagnosis standards were established to study the inner ear malformations [7].

The cochlear duct length was measured using the formula CDL = 4.16A - 3.98, where CDL means the cochlear duct length, and A was calculated by measuring the distance between the round window and the farthest point on the basal turn in the axial section. The minimum length of a normal cochlea is 25 mm. The cochlear height, defined as the measurement from the midpoint of the basal turn to the midpoint of the apical turn, was taken perpendicular to the axes of the cochlear lumens in the coronal view. Normal CHs are 5.2 mm (4.3-6.1 mm) in females and 5.3 mm (4.5-6.2 mm) in males [8].

The vestibular aqueduct width is measured at the midpoint of its course from the vestibule to the opening (operculum) in the posterior cranial fossa. It is considered enlarged if the width is more than 1.5 mm. As the semicircular canal is studied, it can be normal, hypoplastic, absent, or enlarged. On the other hand, as the vestibule is studied, it can be absent, dilated, or normal. Cochlear aperture is measured in axial sections of the HRCT temporal bone and can be normal, hypoplastic (if the width of the bony cochlear nerve canal (BCNC) is less than 1.4 mm), aplastic (when the BCNC is completely replaced by bone or there is no visible canal), or wide (when the width of the BCNC is more than 3 mm) [7].

Data were collected and entered in a Performa, including the patient’s demographic details, audiological findings, and radiological findings, and then the results were analyzed. Data were entered into Microsoft Excel, and statistical analysis was carried out using IBM SPSS Statistics for Windows, Version 27 (Released 2020; IBM Corp., Armonk, New York, United States). Categorical variables were presented as frequency and percentage. The prevalence of inner-ear anomalies was estimated. Correlation between the inner-ear anomaly and other factors was done using the chi-square test.

## Results

**Figure 1:**
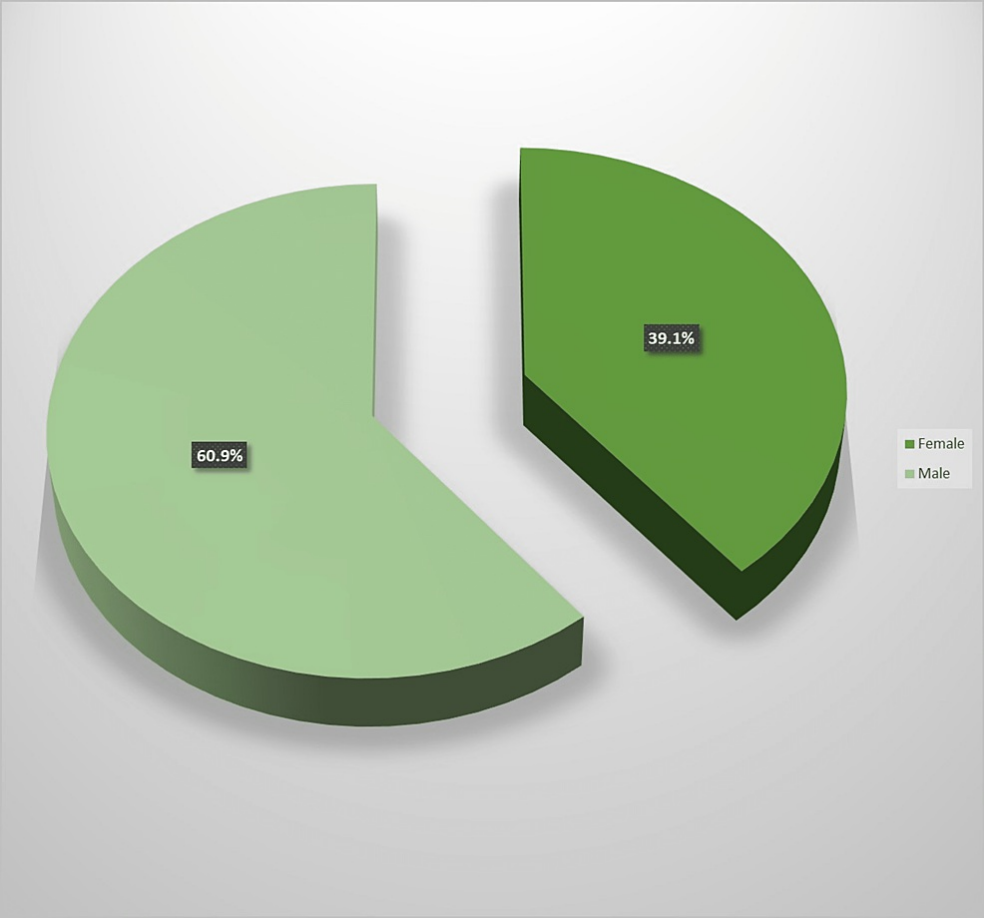
Gender distribution among cases

Forty-six children with congenital SNHL were included in the study; 28 (60.9%) of the participants were males, and 18 (39.1%) of the participants were females (Figure 1).

Thirty-five (76.1%) participants had no family history of hearing loss, and 11 (23.9%) participants reported having a family history of hearing loss with 54.5% in the second degree and 45.5% in the first degree. Consanguineous marriage was reported by 16 (34.7%) participants with 93.8% in the second degree and 6.3% in the first degree. Delay in language development was seen in all participants with hearing loss. In addition, gross motor, fine motor, and social development were also delayed in three (6.5%) of the participants.

Details on birth history showed that two (4.3%) of the participant’s mothers had fever during pregnancy and no mothers had any miscarriage. Forty-three (93.5%) had term delivery, and one (2.2%) of each had an early term, post-term, and pre-term. The majority, 38 (82.6%), were of birth order one followed by five (10.9%) with second-order birth, two were third-order, and one with sixth-order birth. In addition, 38 (82.6%) had normal birth weight, six (13%) had low birth weight, and two (4.3%) had very low birth weight. With regard to the type of delivery, 38 (82.6%) had normal vaginal deliveries followed by seven (15.2%) cesarean section, and one (2.2%) had vacuum-assisted delivery. Seven (15.2%) had a history of NICU admission. The majority, 39 (84.8%) patients, showed a duration of hospital stay of < five days, four (8.7%) with > 10 days, and three (6.5%) with five to ten days.

No participants reported a history of peripartal complications and jaundice. The majority, 44 (95.7%) participants, claimed to be of low socioeconomic status, which meant they were below-poverty-line cardholders. The majority of the patients had a normal general and systemic examination finding. Two (4.3%) patients had microtia-one had bilateral grade three while the other had grade three on right and left grade-four microtia.

There was no child identified with any known syndrome associated with SNHL on examination. However, on further evaluation, one participant had a prolonged QT interval on the electrocardiogram test, which is known to occur in Jervell Lange Nielsen syndrome.

Participants had varying degrees of hearing loss ranging from moderate to profound (Table 1).

**Table 1:** Degree of hearing loss

Degree	Right		left		Total	
	Numbers	Percent	Numbers	Percent	Numbers	Percent
Moderate	1	2.2			1	1.08
Moderately severe	2	4.3	3	6.5	5	5.4
Severe	19	41.3	21	45.7	40	43.4
Profound	24	52.2	22	47.8	46	50
Total	46	100.0	46	100.0	92	100

The prevalence of anomaly in the cochlea structure is 19.5% (nine participants) (Table 2). The anomalies found in this study are incomplete partition type I, II, and III; cochlear hypoplasia type 1 and type 4; and common cavity. Eight of them had the same anomaly bilaterally, while one participant had a common cavity anomaly in the right ear and an incomplete partition in the left ear.

**Table 2:** Cochlea structure CC- Common cavity, CH1- cochlear hypoplasia type 1, CH4- cochlear hypoplasia type 4, IP1- incomplete partition type 1, IP2- incomplete partition type 2, IP3- incomplete partition type 3.

	Cochlea right		Cochlea left	
	Frequency	Percent	Frequency	Percent
Aplasia	1	2.2	1	2.2
CC	1	2.2		
CH1	1	2.2	1	2.2
CH4	3	6.5	3	6.5
IP1	1	2.2	1	2.2
IP2	1	2.2	2	4.3
IP3	1	2.2	1	2.2
Normal	37	80.4	37	80.4
Total	46	100.0	46	100.0

In females, seven (20%) cochleas had an abnormal cochlear height (Table 3). On the other hand, in males, nine (16%) cochleas had an abnormal cochlear height (Table 3). Overall, the prevalence of the abnormal cochlear height was 17.6% (in 16/91 cochlea). The cochlear height was not measured in the cochlea with a common cavity.

**Table 3:** Cochlear height NA: Not applicable

Gender		Right	Left
		Frequency	Frequency
Female	Below normal	4	3
	Normal	13	15
	NA	1	
	Total	18	18
Male	Below normal	4	5
	Normal	24	23
	Total	28	28

The CDL was less than normal in 10 (10.9%) ears (Table 4). The BCNC was abnormal in three (3.2%) ears (Table 4), and in seven (7.6%) ears, it was not measured due to the anomalous cochlear structure.

**Table 4:** Cochlear duct length and bony cochlear nerve canal CDL: Cochlear duct length; BCNC: bony cochlear nerve canal, NA: not applicable

		Right		Left	
		number	percentage	number	percentage
CDL	Normal	41	89.1	41	89.1
	Abnormal	5	10.9	5	10.9
BCNC	Normal	40	87.0	42	91.3
	Abnormal	2	4.3	1	2.2
	NA	4	8.7	3	6.5

 The vestibule was normal in 43 (93.5%) participants and dilated in 39 (6.5%) of them (Table 5).

**Table 5:** Vestibule

	Right		left	
	Frequency	Percent	Frequency	Percent
Dilated	3	6.5	3	6.5
Normal	43	93.5	43	93.5
Total	46	100.0	46	100.0

The majority of the ears, 42 (93.5%) right-side ears, and 44 (95.6%) left-side ears, had a normal vestibular aqueduct (Table 6). The prevalence of anomaly in the vestibular aqueduct is three (6.5%) on the right side and two (4.4%) on the left side (Table 6). A participant who had cochlea with bilateral incomplete partition type II had a bilateral enlarged vestibular aqueduct. One participant with bilateral incomplete partition type I had right vestibular aqueduct aplasia. One participant with bilateral cochlear aplasia also had bilateral vestibular aqueduct aplasia.

**Table 6:** Vestibular aqueduct

	Right		left	
	Frequency	Percent	Frequency	Percent
Aplasia	2	4.3	1	2.2
Enlarged	1	2.2	1	2.2
Normal	42	93.5	44	95.6
Total	46	100.0	46	100.0

The semicircular canal was normal in the majority of the participants (right, 44 (95.7%), left, 45 (97.8%)), and one had aplasia on the right ear with a normal left ear, and one had bilateral hypoplasia (Table 7).

**Table 7:** Semicircular canals

	Right		left	
	Frequency	Percent	Frequency	Percent
Aplasia	1	2.2		
Hypoplasia	1	2.2	1	2.2
Normal	44	95.7	45	97.8
Total	46	100.0	46	100.0

**Figure 2:**
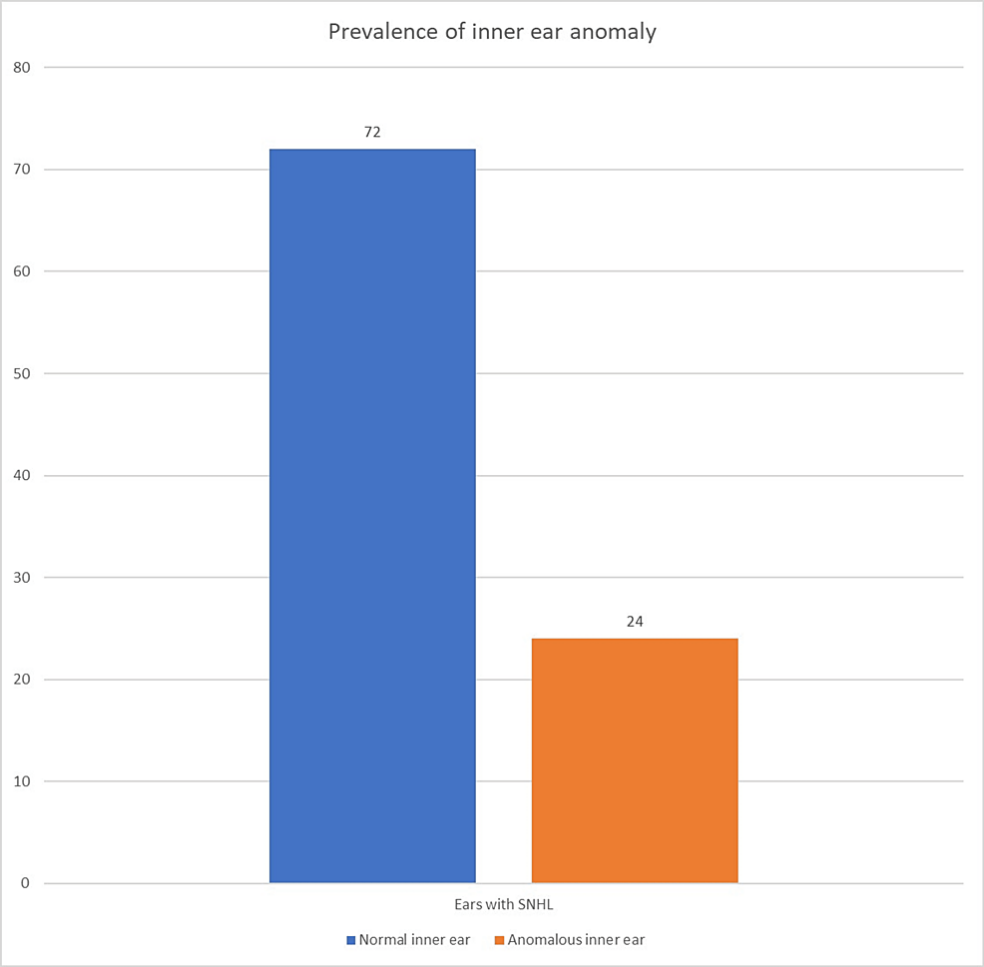
Prevalence of inner-ear anomaly SNHL: Sensorineural hearing loss

Figure 2 shows the prevalence of overall inner-ear anomaly. Any ear with an abnormal cochlear structure, abnormal cochlear height, abnormal cochlear duct length, abnormal vestibule, abnormal vestibular aqueduct, or abnormal semicircular canals was considered as anomalous inner ears. Table 8 shows the prevalence of anomalous inner ear parts in the study’s participants. Out of the 92 inner ears studied, 24 (26.1%) inner ears had anomalies, 22 (23.9%) cochleas were abnormal, six (6.5%) vestibules were abnormal, five (5.4%) vestibular aqueducts were abnormal, and three (3.2%) semicircular canals were abnormal.

**Table 8:** Prevalence of anomalous inner ear parts

	Number of ears	Percentages
Cochlea (structure, height, length)	22	23.9%
Vestibule	6	6.5%
Vestibular aqueduct	5	5.4%
Semicircular canals	3	3.2%

**Figure 3:**
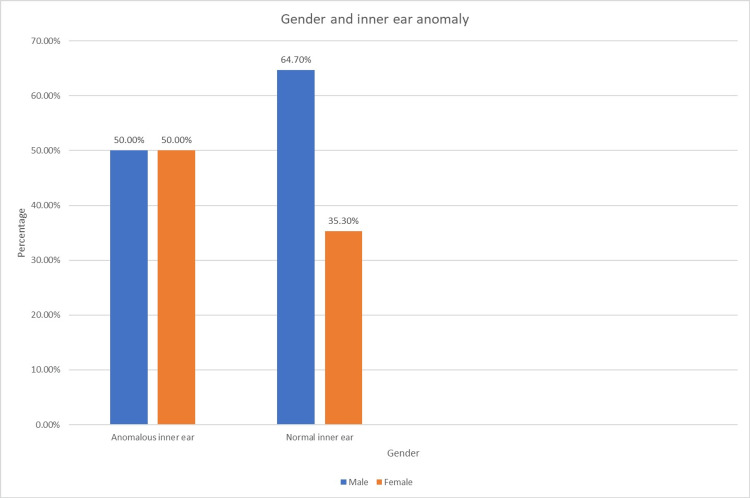
Association of anomalous inner ear and gender

The correlation between the inner-ear anomaly in the participants and gender was analyzed using the chi-square test (Table 9 and Figure 3).

**Table 9:** Association of anomalous inner ear and gender

	Male	Female	Total
Anomalous inner ear	6	6	12
Normal inner ear	22	12	34
	28	18	46

**Figure 4:**
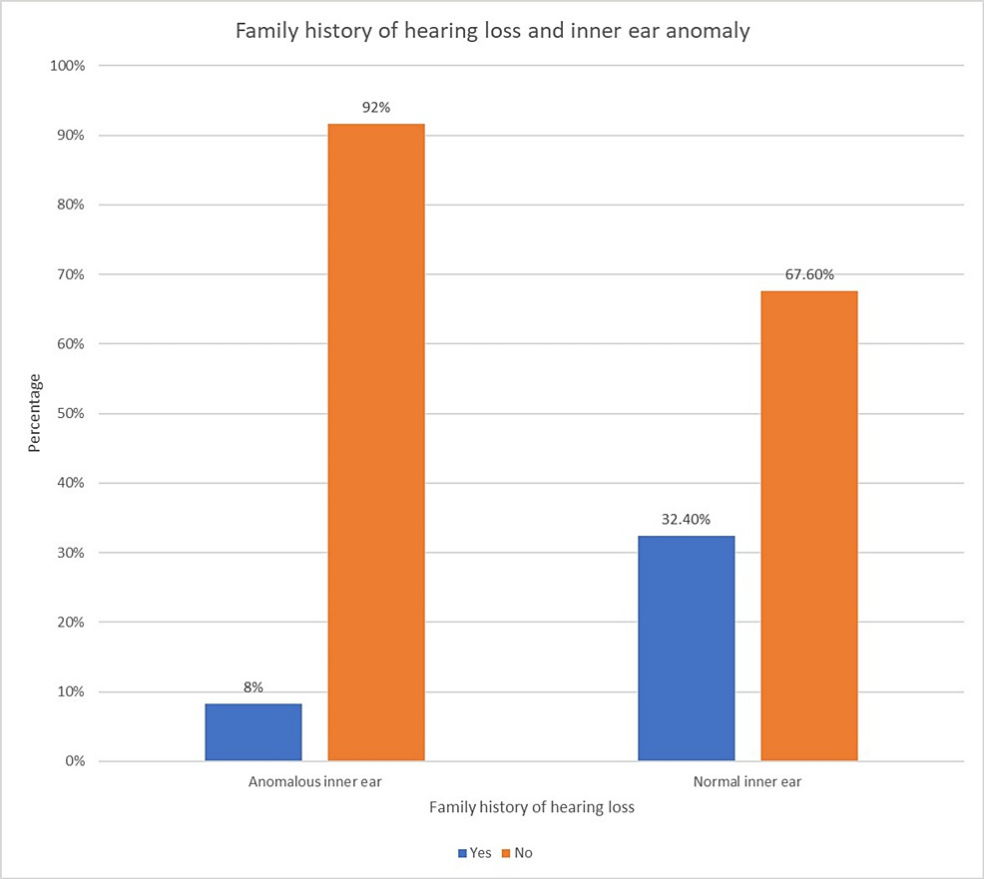
Association of anomalous inner ear and family history of hearing loss

The correlation between the inner-ear anomaly in the participants and family history of hearing loss was analyzed using the chi-square test (Table 10 and Figure 4).

**Table 10:** Association of anomalous inner ear and family history of hearing loss

	Family history of hearing loss	No family history of hearing loss	Total
Anomalous inner ear	1	11	12
Normal inner ear	11	23	34
	12	35	46

**Figure 5:**
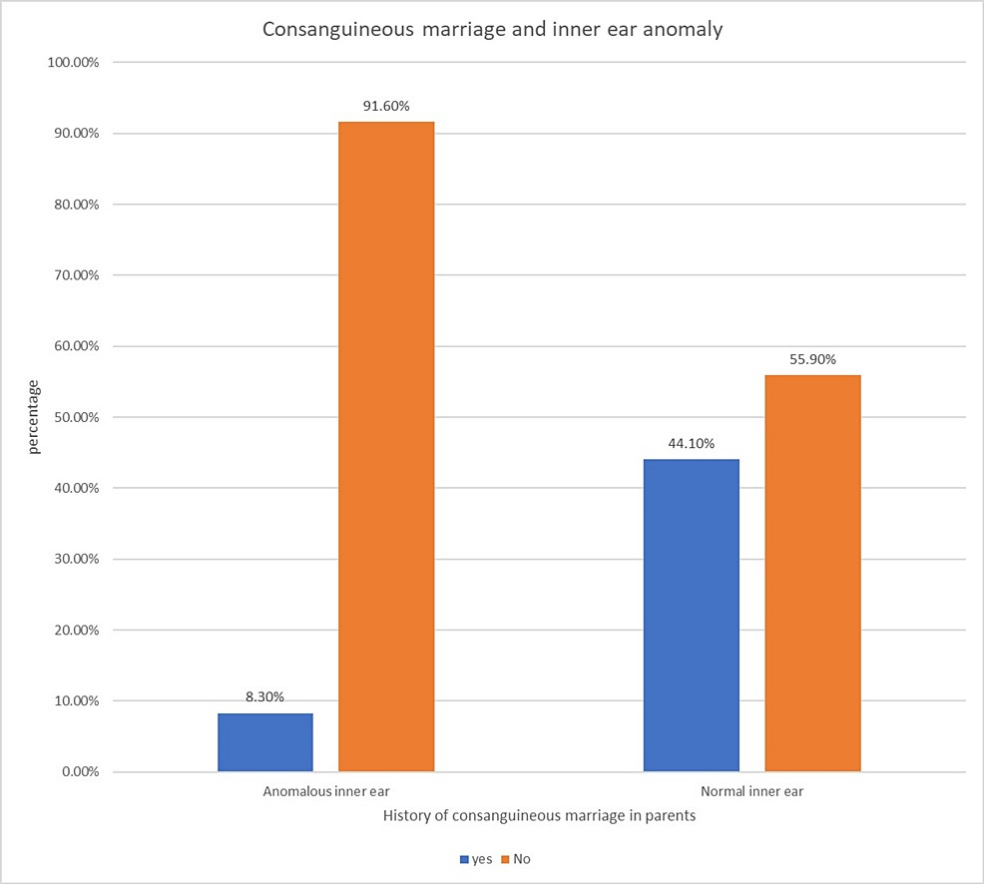
Association of anomalous inner ear and consanguineous marriage

The correlation between the inner-ear anomaly in the participants and their family history of consanguineous marriage was analyzed using the chi-square test (Table 11 and Figure 5). 

**Table 11:** Association of anomalous inner ear and consanguineous marriage

	Consanguineous marriage in parents	No consanguineous marriage in parents	Total
Anomalous inner ear	1	11	12
Normal inner ear	15	19	34
	16	30	46

**Figure 6:**
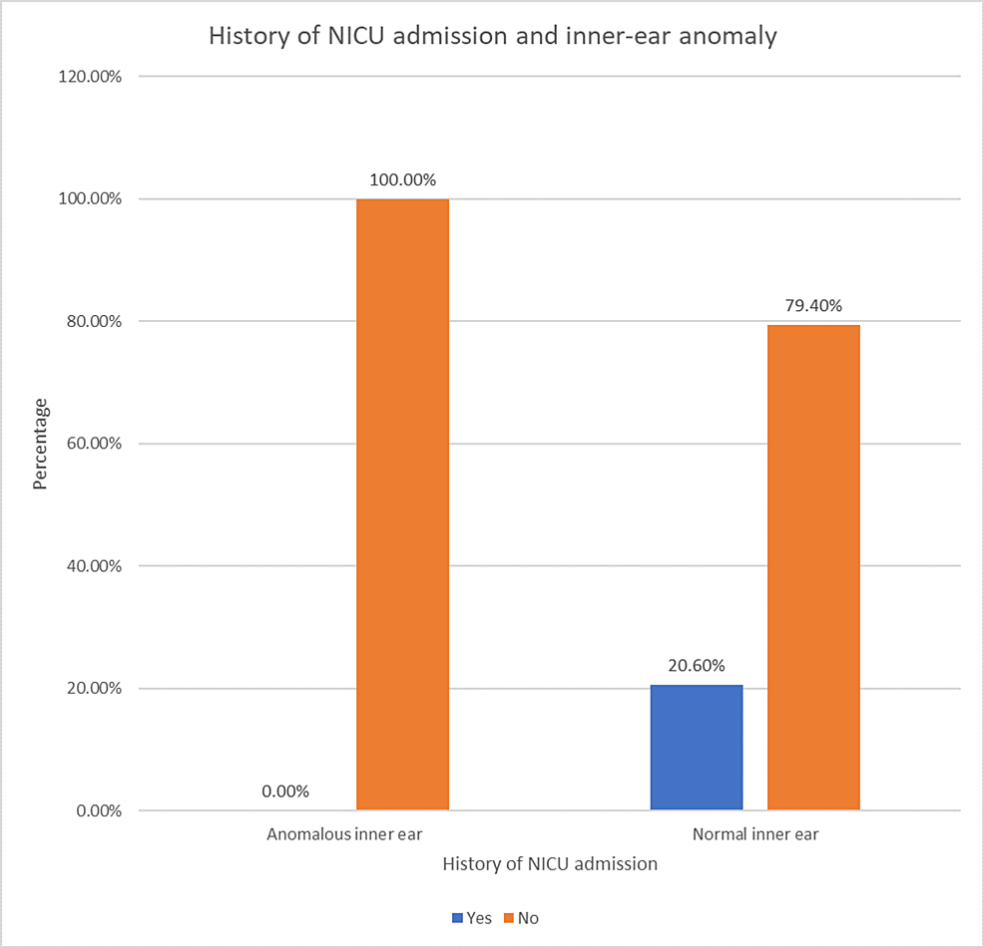
Association of anomalous inner ear and history of NICU admission

The correlation between the inner-ear anomaly in the participants and their history of NICU admission was analyzed using the chi-square test (Table 12 and Figure 6). 

**Table 12:** Association of anomalous inner ear and history of NICU admission

	History of NICU admission	No history of NICU admission	Total
Anomalous inner ear	0	12	12
Normal inner ear	7	27	34
	7	39	46

**Figure 7:**
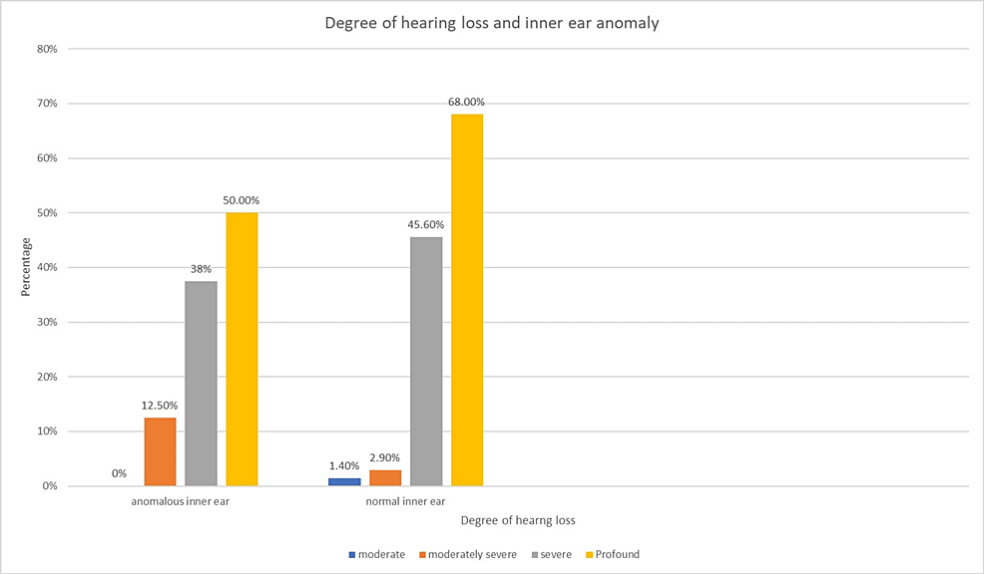
Association of anomalous inner ear and degree of hearing loss

The correlation between the inner-ear anomaly in the participants and degree of hearing loss was analyzed using the chi-square test (Table 13 and Figure 7).

**Table 13:** Association of anomalous inner ear and degree of hearing loss

	Moderate	Moderately severe	Severe	Profound	
Anomalous inner ear	0	3	9	12	24
Normal inner ear	1	2	31	34	68
	1	5	40	46	92

There was no significant association except for a history of consanguineous marriage that had a negative association with the prevalence of inner-ear anomaly in those patients with congenital SNHL with a p-value of 0.015.

## Discussion

**Figure 8:**
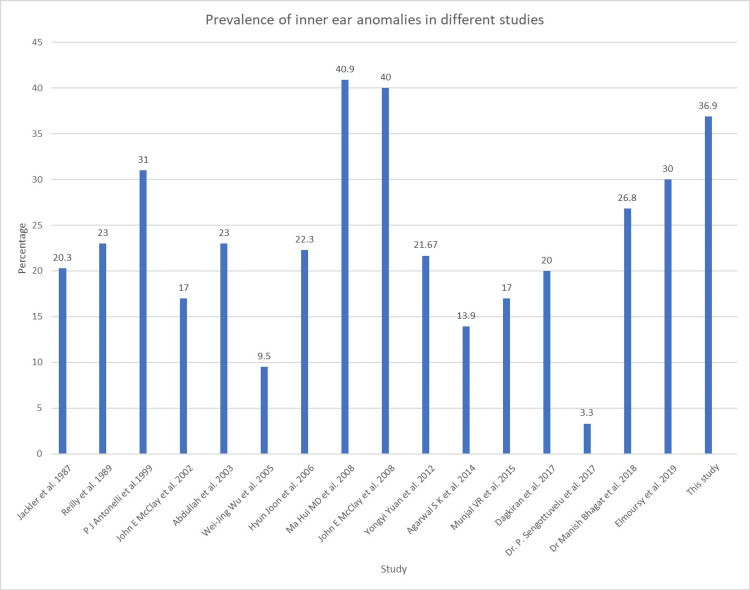
Comparison of this study with studies in the literature Jackler et al. 1987 [5], Reilly et al. 1989 [9], Antonelli et al. 1999 [10], McClay et al. 2002 [11], Abdullah et al. 2003 [12], Wu et al. 2005 [6], Shim et al. 2006 [13], Ma et al. 2008 [14], McClay et al. 2008 [15], Yuan et al. 2012 [16], Agarwal et al. 2014 [1], Munjal et al. 2015 [17], Dağkıran et al. 2017 [18],  Sengottuvelu et al. 2017 [2], Bhagat et al. 2018 [19], Elmoursy et al. 2019 [20]

In this study, a total of 46 children (92 ears) with bilateral congenital SNHL participated. Referring to the classiﬁcation criteria of inner-ear malformations by Jackler and Sennaroglu, diagnosis standards were established to study the inner-ear malformations [7,8]. Out of the 12 children with an abnormal inner ear, two (16.7%) had a unilateral abnormal inner ear, and the remaining 10 (83.3%) had bilateral inner-ear abnormality. Out of the 92 inner ears studied, 24 (26.1%) inner ears had anomalies; 26.08% of cases had inner malformations, which is approximately similar incidence in other studies. The prevalence of inner-ear anomalies in previous studies has been summarized in Figure 8. 

In this study, there was no significant association between inner-ear anomalies and gender or family history or history of NICU admission or degree of hearing loss. However, a history of consanguineous marriage and the prevalence of inner-ear abnormalities were significantly negatively associated. No participants reported a history of peripartal complications and jaundice which are known risk factors associated with hearing loss.

In addition, in this study, all the patients with inner-ear anomalies had cochlear anomalies. Jackler et al. found 76% cochlear abnormalities [5]. Dağkıran et al. found cochlear abnormalities in 13% of their series [18]. Vestibular abnormality was seen in three (6.5%) patients, all of whom had dilated vestibules, whereas a study done by Wu et al. found 3% vestibular abnormalities [6].

Moreover, three (6.5%) patients in this study had vestibular aqueduct abnormality, of which two (66.6%) patients had dilated vestibular aqueduct and one (33.3%) with absent vestibular aqueduct. Of the 46 cases, two (4.3%) patients had semicircular canal abnormalities involving all three semicircular canals. Dağkıran et al. found posterior semicircular canal malformation in 7.3% of their patients, lateral semicircular canal in 7.4%, and superior semicircular canal aplasia/hypoplasia in 5.9% of their patients [18].

A study done by Vishal et al. showed that knowledge of radiological abnormalities in temporal bone or brain can provide reasonable help in predicting the auditory perception outcome [21]. Hence, it is important to know the radiological abnormalities before a cochlear implant.

Limitations

The study was done on a small population of only 46 participants. Results could have better coverage if a larger sample size was studied and for a longer duration. There are limitations in the utilization of CT in identifying an enlarged vestibular aqueduct, and MRI is the gold standard for the same procedure. Middle-ear and external-ear anomalies and other structures, like facial nerves, were not analyzed in this project. A detailed analysis of etiology for congenital SNHL like herpes, CMV, toxoplasmosis, rubella, and genetics was not done as there was no availability of laboratory tests. Finally, a detailed evaluation for the identification of syndromes via ECG/2D Echo/abdominal USG or other methods of investigation could not be done in all participants.

## Conclusions

HRCT scanning of the temporal bone could provide detailed information of pathology of the inner ear in congenital SNHL, which can help in planning better, the surgery for cochlear implantation and understanding the prognosis. The prevalence of inner-ear anomaly found in this study was approximately similar to previous studies. The unusual finding of this study is the negative correlation between the history of consanguineous marriage and inner-ear anomaly.

## Figures and Tables

**Figure 9:**
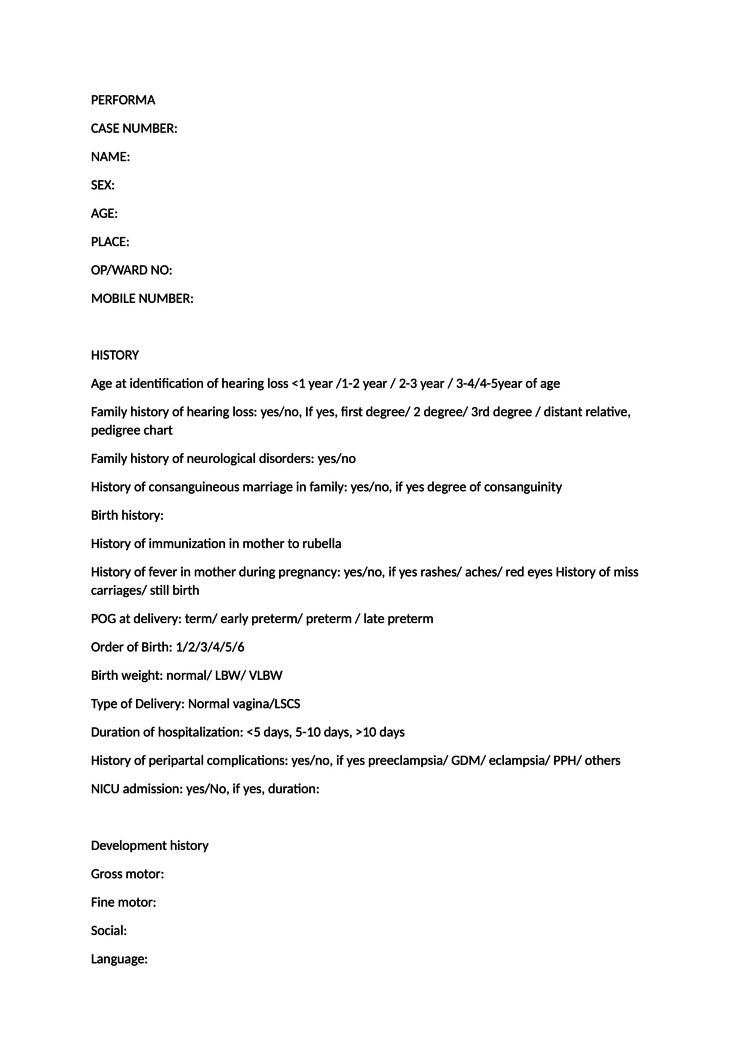
Performa, page 1

**Figure 10:**
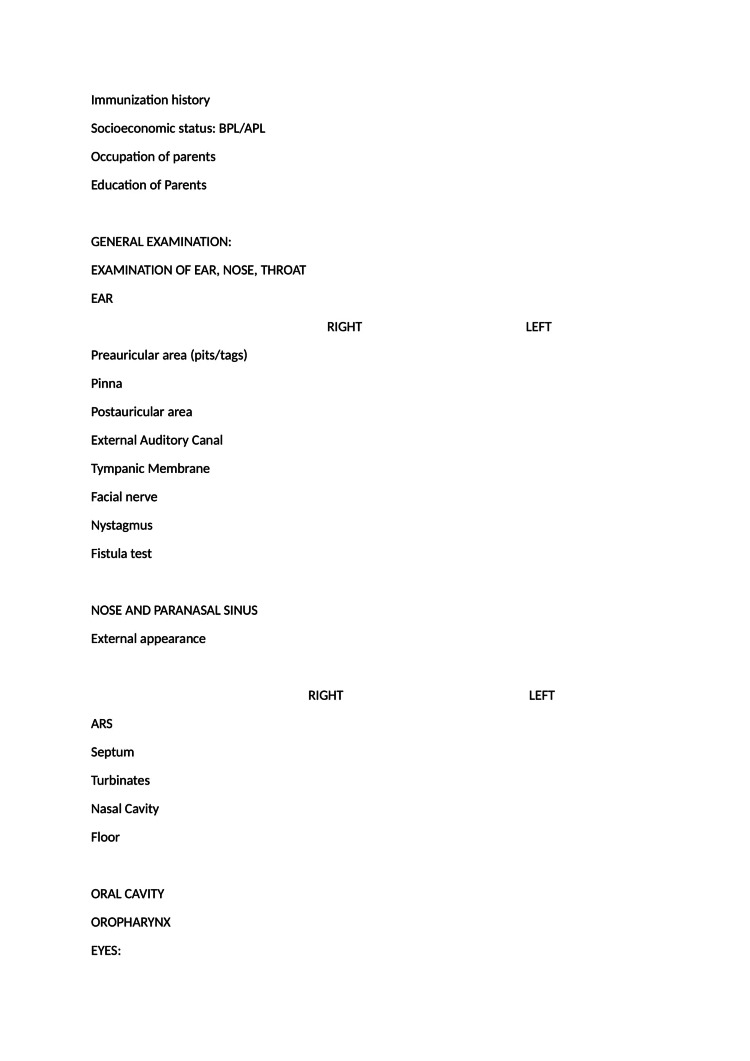
Performa, page 2

**Figure 11:**
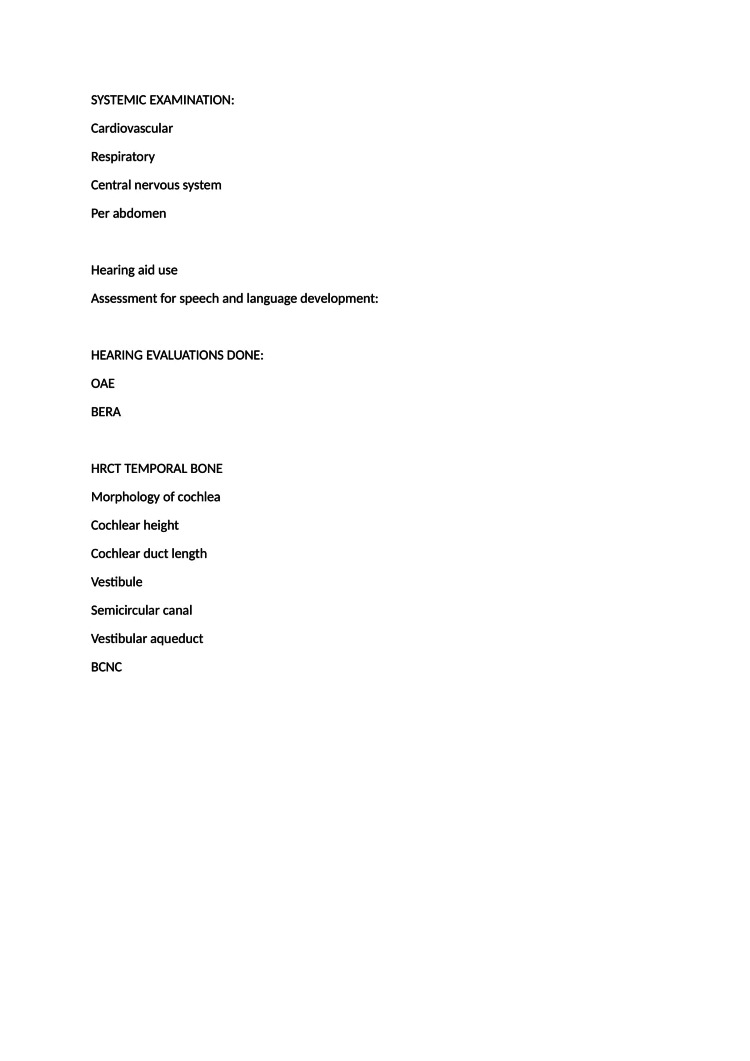
Performa, page 3
